# Prevalence of suicidal behavior in a northeastern Mexican border population during the COVID-19 pandemic

**DOI:** 10.3389/fpsyg.2022.984374

**Published:** 2023-01-10

**Authors:** Karla Villarreal Sotelo, Fabiola Peña Cárdenas, Benito Zamorano González, Cynthia Marisol Vargas Orozco, Ignacio Hernández Rodríguez, Carolina Landero Pérez

**Affiliations:** ^1^Postgraduate Department, UAM-Reynosa Aztlán, Universidad Autónoma de Tamaulipas, Reynosa, Tamaulipas, Mexico; ^2^Postgraduate Department, UAM-Matamoros, Universidad Autónoma de Tamaulipas, Matamoros, Tamaulipas, Mexico

**Keywords:** suicide, COVID-19, suicidal behavior, Mexican, suicidal ideation

## Abstract

**Introduction:**

Before the pandemic, suicide was already considered a global public health problem. The outbreak of COVID-19, a coronavirus-related infectious disease, began to impact people's physical and mental health. The factors that either contribute to or mitigate this risk need to be better understood, and this can only be accomplished through research. Therefore, this study aimed to study the prevalence of suicidal ideation and behavior in Tamaulipas, Mexico, during the COVID-19 pandemic.

**Methods:**

A quantitative, descriptive, and cross-sectional study was conducted. The sample consisted of 659 participants, of whom 194 (29.5%) were men and 465 (70.5%) participants were oldwomen, ranging in age between 16 and 68 years (M = 22.56, SD = 7.26). An adapted version of the Spanish version of the Columbia Suicidal Severity Rating Scale was used to assess the seriousness of suicidal ideation and behavior.

**Results:**

The higher rates of suicidal indicators were suicidal ideation with “wish to dead” (39.9%), while the lower was suicidal ideation with a specific plan (8.2%). A total of 18.2% of participants reported “suicidal attempts before COVID-19,” of whom 40% reported “suicidal attempts in the last 3 months.” Suicidal behavior rates were lower: 13.7% of participants reported “non-specific preparatory behavior” and 13.3% reported “actual suicide attempts.” Women were more likely than men to exhibit almost all indicators of suicidal ideation and behavior (OR = 1.63–2.54; 95% CI = 1.11–2.41, 1.76–3.68), as well as confinement (OR = 2.60; 95% CI = 1.73–3.91). Confinement for more than 40 days (OR = 0.55–0.66; 95% CI = 0.40–0.75, 0.47–0.93) and knowing a person infected with COVID-19 (OR = 1.57–2.01; 95% CI = 1.02–2.42, 1.20–3.34) were associated with a higher risk of exhibiting several suicidal indicators and having previously attempted suicide.

**Conclusion:**

Being a woman, knowing a person infected with COVID-19, and being confined, especially for longer than 40 days, are all risk factors for suicidal ideation. Therefore, intervention programs are needed to reduce the suicide risk prevalence, especially these days because of the influence of the pandemic, and should be primarily focused on those who present the risk factors associated with suicidal behavior identified in this study.

## Introduction

A large number of authors have contributed to the generation of knowledge on mental health, especially on suicidal ideation during the pandemic (Fitzpatrick et al., 2020; Longobardi et al., 2020; Cheung et al., 2021; Nomura et al., 2021; Smalley et al., 2021). All of them intend to understand the impact of COVID-19 on suicidal thoughts and behaviors, personality traits, and other psychosocial aspects. The first case of COVID-19 was reported in Wuhan, China, in December 2019 (Nomura et al., 2021). Afterward, the cases continued to increase exponentially. Faced with mounting cases, the World Health Organization (WHO) declared a pandemic in March 2020, which marked the beginning of a series of measures and strategies to prevent its spread. However, in many countries, the crisis still persists due to the daily increase in infections and, even worse, deaths, which are close to four million globally (Aragón-Nogales et al., 2019; Longobardi et al., 2020; World Health Organization, 2020).

The implementation of prevention measures has caused a drastic change in daily activities (Odone et al., 2020; Lee et al., 2022; Rahman et al., 2022). Most of these measures consist of maintaining physical isolation and social distancing, actions that, to this day, are effective methods to reduce the possibility of further transmission of the infection. Work-at-home and virtual work has disrupted daily life. The world faced temporary closures of schools and businesses, as well as all activities considered non-essential, which resulted in the loss of many people's sources of income. The population experienced a drastic change in their daily lives due to the pandemic containment measures. Under this context, some people lost their jobs, others stopped seeing their families due to the “stay-at-home” measures, and many others suffered from anxiety due to the fear of becoming ill with COVID-19 and ending up in an intensive care ward or, even worse, dying (Devitt, 2020; Ammerman et al., 2021).

The indirect consequences of the prevention actions began to manifest in the health of many people who presented some symptoms related to their mental health, such as stress, anguish, feelings of loneliness, and high levels of suicidal ideation, with adolescents and young adults being the most vulnerable population (Kiuchi et al., 2020; Nomura et al., 2021). Studies around the world reported a significant increase in psychological distress, COVID-19 anxiety syndrome, affective symptoms, allostatic load, and fatigue, which have been observed across different populations. Differences in mental health were described based on the country or culture of origin, age, sex, occupation, community, or clinical samples (Zhang et al., 2020; Brailovskaia et al., 2021; Mansueto et al., 2021, 2022).

Previous studies exploring the psychological impact of the COVID-19 population's mental health stated that how people perceive and deal with the COVID-19 situation can impact their mental and physical health (Galea et al., 2020; Salari et al., 2020). Some of them have focused on external factors such as perceived social support (Huang et al., 2021), stress related to the type of occupation, such as healthcare workers (Mansueto et al., 2021), or the use of social media (Brailovskaia et al., 2021). For example, in Germany and Italy, Brailovskaia et al. (2021) stated that social media use was positively linked to stress and burden caused by COVID-19 because social media is used as a COVID-19 information source.

Other studies focused on individual psychological resources that could act as risk or protective factors to deal with COVID-19-related consequences. In China, the study by Zhang et al. (2020) discovered that resilience and positive coping were protective factors for depressive, anxiety, and stress symptoms in junior high and high school students, whereas negative coping was a risk factor. For example, Zhang et al. (2020) pointed out that individual factors such as coping strategies are crucial to dealing with COVID-19-related consequences. A longitudinal study pointed out that, while some individuals experience the restrictions on daily life as a heavy burden, others adapt to the situation and try to make the best of it (Brailovskaia and Margraf, 2020).

The previously daid, or the previously exposed with regard to COVID-19 contingency has caused drastic changes in health statistics, especially those referring to mortality rates (Longobardi et al., 2020), forcing professionals and researchers to find ways to assess and intervene to address the mental health problems stemming from the sanitary crisis (Kar et al., 2020; Kahil et al., 2021). With regard to suicide, research showed that, for a person to commit suicide, it requires a “perfect storm” that includes the presence of some mental illness, some genetic predisposition, family history, the presence of certain personality traits, the consumption and abuse of alcohol or drugs, and some problematic events that cannot be addressed (Devitt, 2020; Reger et al., 2020; Kahil et al., 2021). For example, studies pointed out that patients with higher total scores on affectively dysregulated temperaments are more likely to have higher levels of hopelessness, which is an important predictor of suicidality (Serafini et al., 2012).

Recent research showed that suffering from an illness that could prove burdensome for the family, like COVID-19, can raise feelings of anxiety, sorrow, and stress, leading some to consider suicide as a means to escape their moral and financial responsibilities to their loved ones (Devitt, 2020). For this reason, disorders related to mental health, particularly those related to suicide, represent a global public health problem affecting adolescents and young adults, which has been considered the second leading cause of death in these age groups in recent years (Han et al., 2020; Penninx et al., 2021).

Fear of the COVID-19 disease, the vulnerability of individuals, and a history of depressive symptoms are considered significant risk factors for suicidal ideation (Saricali et al., 2020). Negative lifestyles such as smoking, drinking, and drug use, which increased during the pandemic, also played a role (Nomura et al., 2021). On the other hand, staying at home has led to a mental burden that increases levels of depression and anxiety in the young, elderly, and fragile populations and cannot be ignored, making it a risk factor for suicidal ideation and behavior (Sayeed et al., 2020; Nomura et al., 2021; Rahman et al., 2021; Pathirathna et al., 2022).

Nevertheless, despite an increasing number of studies conducted worldwide, a gap in knowledge exists about Mexican studies. In the particular case of Mexico, the National Institute of Statistics and Geography (INEGI) described an increase in suicide rates between 2017 and 2018. The cases of self-inflicted deaths per 100,000 inhabitants in 2017 reached a value of 5.2, while in 2018, it was 5.4 (INEGI, 2020). The present study presented data obtained from the project “Suicide in times of COVID-19,” conducted under the sponsorship of the Universidad Autonoma de Tamaulipas. The participants in the study were located in the northeastern region of Mexico, which is characterized by social vulnerability, social violence, migration, and social marginalization, mainly due to its proximity to the United States. The present study aimed to report the prevalence of suicidal ideation and behavior related to COVID-19 in the population along the northeastern border of Mexico. To that end, our first specific objective was to determine the prevalence of suicidal ideation and behavior in the people of the northeastern border of Mexico both before and during the COVID-19 pandemic. The second aim was to analyze the suicidal ideation and behavior associated with the risk factors of contagion from COVID-19. Finally, we sought to analyze the suicidal ideation and behavior associated with confinement during the COVID-19 pandemic. Based on the previous literature review, we hypothesized that, during the COVID-19 pandemic, there might be an increased prevalence of suicidal ideation and behavior in individuals on the northeastern border of Mexico. The second risk factor for suicidal ideation and behavior is COVID-19 contagion. Finally, confinement is a risk factor for suicidal ideation and behavior.

## Materials and methods

### Participants

In this study, a quantitative, descriptive, and cross-sectional study was conducted. The participants of the present study were an open population from the northeastern border of Mexico. The sample consisted of a total of 659 participants, of whom 194 (29.5%) were men and 465 (70.5%) were women, ranging in age from 16 to 68 years (M = 22.56, SD = 7.26). Participants were selected by a non-probabilistic convenience sampling procedure. Due to the pandemic lockdown measures, virtual sampling was combined with an online questionnaire because of the inability to reach the target population face-to-face. The online questionnaire was distributed by the study team's social media channels using a snowball technique. Participants were evaluated in September 2020, 6 months after the first cases of COVID-19 were detected in Mexico, and were recommended for population confinement and other sanitary measures to contain the first wave of the pandemic.

### Procedure

As previously stated, the process for data collection due to the health contingency was developed through an electronic questionnaire *via* Google Forms because of the inability to reach the target population face-to-face. A snowball procedure was followed to distribute the online survey through the research team's social media and digital platforms. This procedure was selected since the use of virtual sampling combined with an online survey has proven to be a reliable tool to guarantee an increase in the sample size and its representativeness of hard-to-reach target populations, as presented in the case of the COVID-19 lockdown. No compensation was provided to the participants. The information was processed to calculate descriptive statistics and reliability coefficients and determine the odds ratios (OR) at a 95% confidence interval using SPSS v22 statistical software.

### Instrument

A questionnaire requesting sociodemographic data and behaviors related to confinement was designed specifically for the COVID-19 pandemic.

The first section included questions regarding their sociodemographic characteristics, such as sex, age, marital status, living with, schooling, occupation, and place of residence, to assess demographic variables.

The second section included questions regarding COVID-19 status and confinement. This section asked participants to indicate whether they had been confined and whether they had been infected with COVID-19 at any point (current or in the past), as well as whether they knew someone who had been infected with the virus. The items included were as follows: “Have you been infected by COVID-19?,” “Are you infected by COVID-19?,” “Has anyone you know been infected with COVID-19?,” and “Has anyone you know died of COVID-19?” Regarding personal confinement, the question included was, “Have you been in confinement?” with dichotomous answer options: “yes” or “not.” To assess the time of confinement, the question included was, “If your previous answer was yes, for how long?” It included three answer options: “ <14 days,” “14–39 days,” and “more than 40 days.”

In addition, an adapted version of the Spanish version of the Columbia Suicidal Severity Rating Scale was used to assess the seriousness of suicidal ideation and behavior (Sp-CSSR-S). CSSRS is a well-known measure in suicide research because of its construction and validation in the English language (Posner et al., 2010, 2011); it has been translated into multiple languages and adapted to various cultures. The translated version into Spanish has been validated with Spanish-speaking psychiatric outpatients. It is a reliable and valid instrument for assessing suicidal ideation and behavior in daily clinical practice and research settings (Al-Halabi et al., 2016). Since the adaptation of an instrument is considered new, for the analysis of the instrument's reliability, the coefficient of Cronbach's alpha was calculated in our sample. The instrument presented a Cronbach's alpha of 0.92, which is considered high and, therefore, adequate to continue with the study.

The items evaluated ideas and behaviors in increasing order of intensity and risk. The general instruction was modified to ask whether these ideas and behaviors had occurred during the COVID-19 health emergency. Because the CSSRS is not a time-sensitive measure, we included three items to assess the period preceding the COVID-19 health emergency and one to assess the 3 months preceding the assessment. We described the items included below:

Suicidal ideation assessed by items 1–5 included ideas related to death, with or without a specific plan, and a desire to commit suicide, but there was no behavioral activation.

(a) The wish to be dead, in which the person confirmed that they had had ideas related to wishing to be dead or to no longer live, or the wish to fall asleep and not wake up, was asked if the participants answered “Yes” to Question 1: “Have you wished to be dead or to be able to fall asleep and not wake up?”.
(b) Non-specific suicidal ideation, which explored general, non-specific thoughts regarding the desire to end one's life/suicide without ideas about how to do it, was explored in Question 2: “Have you actually had the idea of committing suicide?”.
(c) Suicidal ideation without any method, which assessed whether the person had the idea of committing suicide and had thought about how they would do it, was explored in Item 3: “Have you thought about how you would carry this out?”.
(d) Suicidal ideation (no specific plan), in which the person had the idea and thought about carrying out their plan, was assessed with Question 4: “Have you had these ideas and, to some degree, intended to carry them out?”.
(e) Suicidal ideation with a specific plan, that is, ideas of taking one's own life are presented with details of the plan partially or fully elaborated, and the participant has the intention of carrying out this plan, as assessed by Question 5: “Have you started to elaborate or have you elaborated the details of how to commit suicide?”.

Determination of the temporal spaces of suicidal ideation and behavior (Questions 6–8): These items were included to assess suicidal ideation and behavior before and during the COVID-19 pandemic.

(f) Previous suicide attempts, where the person had already moved from idea to action, were explored with Question 6: “Have you ever done anything, started anything, or prepared to do anything to end your life?”.
(g) Using the previous questions as a reference, Question 7 on the time period was included: “If your previous answer was yes, was this in the last 3 months?”.
(h) Previous suicidal ideation or behavior was explored with Question 8: “Have you had suicidal thoughts or attempts in other periods of your life?”.
(i) Active suicidal behavior was assessed by Questions 9–11. Non-specific preparatory behavior by Question 9: “Have you started planning something to end your life?” At this point, there was active suicidal ideation without any method or plan.
(j) Preparatory behavior with a specific plan by Question 10: “Have you prepared something to end with your life?” At this point, there is active suicidal ideation with a specific plan but not the intent.
(k) Actual suicide attempt by Question 11, “Have you done something to end your life?,” to assess. In this case, the person has already attempted to end their life, which has failed.

### Ethical considerations

The project that supports this manuscript was submitted for evaluation and approval by the Postgraduate and Research Committee of the Autonomous University of Tamaulipas (Universidad Autonoma de Tamaulipas, Grant number: 38-UATINVES20). The study did not include any risk for the participants; however, according to the Declaration of Helsinki and the Code of Health Research in Mexico, online informed consent was requested from the participants in the survey to continue. It explained the study's purpose, the participants' anonymity, the confidential handling of the data, and voluntary participation. At the same time, participants were informed that if they wished to decline participation, they could do so at any time once the survey had begun.

### Statistical analyses

The information from the answered questionnaires was uploaded into a database, which was then edited and analyzed in the Statistical Package for the Social Sciences (SPSS) version 22. Information was generated about the descriptive statistics for demographic data by percentages, mean scores, and standard deviation. To analyze the risk of suicidal indicators associated with sex, COVID-19 and confinement odds ratios (OR) for a 95% confidence interval (CI) were calculated. Significant differences were considered when the confidence intervals did not include the unit. Variables' answers were coded as 0 for “not” and 1 for “yes.” Further, when contrasting by risk factor sex, 0 was used for “men” and 1 for “women.” Similarly, the time of confinement, “40 days,” was coded with 0 and “more than 40 days” with 1. The results can be seen in Tables 1–**6**.

**Table 1:** Demographic data of the participants.

**Variable**	***n***	**%**
**Sex**		
Men	194	29.4
Women	465	70.6
**Marital status**		
Single	552	83.8
Married or in free unión	98	14.9
Divorced	6	0.9
Widow	3	0.5
**Live with**		
Family	560	85
Partner	54	8.2
Alone	34	5.2
Friends	7	1.1
Other	4	0.5
**Schooling**		
Posgrade	50	7.5
Higher education	461	70
High school	135	20.5
Middle school	13	2
**Occupation**		
Empresary/comerciant	16	2.4
Formal employee	192	29.1
Informal employee/temporary worker	116	17.6
Housewife/Not work	19	2.9
Student	316	48
**Place of residence**		
Tamaulipas	613	93
Other	46	7

Source: Data from study survey.

## Results

Table 1, the demographic data of the participants, presents a summary of the main characteristics of the study population. Regarding the sex of the population, 465 women (70.6%) and 194 men (29.4%) were identified. Their marital status identified 552 single participants (83.8%) and 98 married participants (14.9%). Most of the participants lived with their families, 560 (85%), while 54 lived with a partner (8.2%), and 34 lived alone (5.2%). In terms of schooling, 461 were part of the higher education group (70%), followed by 135 high school students (20.5%) and 50 graduate students (7.5%). Regarding occupation, almost half of them were only students: 316 (48%); the rest of the participants did another activity: 192 participants (29.1%) were formal employees, while 116 subjects (17.6%) were informal employees or temporary contract workers. Regarding their place of residence, 613 participants (93%) responded that they reside in Tamaulipas, Mexico.

### Prevalence of suicidal ideation and behavior during the COVID-19 total and by sex

To meet the objective of presenting the prevalence of suicidal ideas and behaviors, these were first analyzed in all participants and later contrasted by sex. Our main findings showed that the most frequent indicators of suicidal ideation were “wish to be dead,” reported by 39.9%, “unspecific suicidal ideation,” reported by 30.5%, “suicidal ideation with any method,” reported by 29.7%, and “suicidal ideation without a specific plan,” reported by almost a quarter of the participants; less frequent were the indicators of suicidal behaviors such as “non-specific preparatory behavior,” reported by 13.7% of the participants, “actual suicide attempts” (13.3%), and 10.2%, “non-specific preparatory behavior.” Nearly a fifth of participants (18.2%) reported “previous suicidal attempts,” of whom 40% reported “suicidal attempts in the last 3 months” and 42.3% had a history of “previous suicidal ideation or attempts” (see Table 2). Then, odds ratios (OR) for a 95% confidence interval (CI) for each suicidal indicator were calculated to determine the risk associated with the sexes. It was considered statistically significant when the 95% CI did not include the value of 1; this identified the group at risk. It was found that the female sex was a risk factor for 8 of the 11 items evaluated: “wish to be death” (OR = 2.15, 95% CI = 1.49–3.09); “unspecific suicidal ideation” (OR = 2.04, 95% CI = 1.36–3.05); “suicidal ideation with any method” (OR = 1.63, 95% CI = 1.11–2.41); “suicidal ideation without a specific plan” (OR = 1.97, 95% CI = 128–3.05); “previous suicidal attempts” (OR = 1.73, 95% CI = 1.07–2.79); “previous suicidal ideation or attempts” (OR = 2.54, 95% CI = 1.76–3.68); “non-specific preparatory behavior?” (OR = 1.84, 95% CI = 1–05–3.24); and “actual suicide attempts” (OR = 2.35, 95% CI = 1.28–4.29). Details of the information can be seen in Table 2: Suicidal ideation and suicidal behavior during COVID-19. Or rates of suicidal ideation and suicidal behavior.

**Table 2:** Prevalence of suicidal ideation and behavior during contingency by COVID-19 total and by sex.

**Suicidal behavior**	**Total**					**Sex**								**OR**	**CI**
						**Women (*****n*** = **465)**				**Men (*****n*** = **194)**					
		**Yes**		**No**		**Yes**		**No**		**Yes**		**No**			
	***N***	**Frec**.	**%**	**Frec**.	**%**	**Frec**.	**%**	**Frec**.	**%**	**Frec**.	**%**	**Frec**.	**%**		
1. Wish to be dead	656	262	39.9	394	60.1	209	45	255	55	53	27.6	139	72.4	2.15^***^	1.49–3.09
2. Unspecific suicidal ideation	653	199	30.5	454	69.5	160	34.6	303	65.4	39	20.5	151	79.5	2.04^***^	1.36–3.05
3. Suicidal ideation with any method	656	195	29.7	461	70.3	151	32.6	312	67.4	44	22.8	149	77.2	1.63^**^	1.11–2.41
4. Suicidal ideation without a specific plan	656	159	24.2	497	75.8	128	27.6	336	72.4	31	16.1	161	83.9	1.97^**^	1.28–3.05
5. Suicidal ideation with a specific plan	657	54	8.2	603	91.8	37	8	428	92	17	8.9	175	9.1	0.89	0.48–1.62
6. Previous suicidal attempts	656	120	18.2	536	81.7	95	20.5	368	79.5	25	13	168	87	1.73*	1.07–2.79
7. Suicidal attempts in the last 3 months	120	48	40	72	60	39	41.1	56	58.9	9	36	16	64	1.23	0.49–3.08
8. Previous suicidal ideation or attempts	653	276	42.3	377	57.7	225	48.5	239	51.5	51	27	138	73	2.54^***^	1.76–3.68
9. Non-specific preparatory behavior	626	86	13.7	540	86.3	69	15.7	371	84.3	17	9.1	169	90.9	1.84*	1.05–3.24
10. Preparatory behavior with a specific plan	629	64	10.2	565	89.8	51	11.5	391	88.5	13	7	174	93	1.74	0.926–3.29
11. Actual suicide attempt	631	84	13.3	547	86.7	70	15.8	372	84.2	14	7.4	175	92.6	2.35^**^	1.28–4.29

Odds ratio, calculated for 95% certainty. When the confidence interval did not include the value 1. p ≤ 0.05;

** p ≤ 0.01;

*** p ≤ 0.001.

### Prevalence of COVID-19 infection and the total number of confinement days, as well as the sex of the participants

Table 3 presents a description of the data regarding COVID-19 infection, the **total number of** confinement days, and, in contrast, by sex. COVID-19 infected 18.8% of the participants, 82.9% knew someone who was infected, and 54.8% knew someone who died from COVID-19. More than four-fifths of the participants reported living in confinement (81.6%). Then, odds ratios (OR) for a 95% confidence interval (CI) for each sex were calculated to determine the risk associated with the sexes. It was considered statistically significant when the 95% CI did not include the value of 1. The questions posed showed that there was no association between these variables, except for the question “Have you been in confinement?” (OR = 2.60, 95% IC = 1.73–3.91); this rate was higher in women (86.2%) than in men (70.6%).

**Table 3:** Prevalence of COVID-19 infection and confinement total, and in contrast by sex.

**Behavior**	**Total**					**Sex**								**OR**	**CI**
		**Women (*****n*** = **465)**				**Men (*****n*** = **194)**									
	***n***	**Yes**		**No**		**Yes**		**No**		**Yes**		**No**			
		**Frec**.	**%**	**Frec**.	**%**	**Frec**.	**%**	**Frec**.	**%**	**Frec**.	**%**	**Frec**.	**%**		
Have you been infected by COVID-19?	659	124	18.8	535	81.6	94	20.2	371	79.8	30	15.5	164	84.5	1.38	0.88–2.17
Actually are you infected by COVID-19?	659	7	1.1	652	98.9	4	0.9	461	99.1	3	1.5	191	98.5	0.52	0.12–2.49
Has anyone you know been infected with COVID-19?	659	546	82.9	113	17.1	390	83.9	75	16.1	156	80.4	38	19.6	1.26	0.82–1.95
Has anyone you know died for COVID-19?	659	361	54.8	298	42.5	265	57	200	43	96	49.5	98	50.5	1.35	0.96–1.89
Have you been in confinement?	659	538	81.6	121	18.4	401	86.2	64	13.8	137	70.6	57	29.4	2.60^***^	1.73–3.91
If your previous answer was YES, for how long?															
Less than 14 days	538	76	14.1			49	12.2			27	19.7				
14–39 days	538	145	27			106	26.4			39	28.5				
More than 40 days	538	317	58.9			246	61.3			71	51.8				

Odds ratio, calculated for 95% certainty. When the confidence interval did not include the value 1.

*** p ≤ 0.001.

### Association between suicidal ideation and behaviors in relation to personal COVID-19 infection

When comparing the rates of suicidal indicators between participants who were ever infected vs. “not” and infected vs. “not,” the odds ratio (OR) was calculated to quantify the association between the possible risk factor and the percentage of each suicidal indicator, and it was considered statistically significant when the 95% CI did not include the value of 1; this identified the groups at risk. Table 4 shows the association between suicidal thoughts and behaviors related to having suffered from a COVID-19 infection. As it can be seen, those infected were only at a higher risk of reporting a “wish to be dead,” with 48.4% of them doing so in contrast to the 38% of participants who were never infected (OR = 1.53, 95% CI = 1.03–2.27).

**Table 4:** Association between suicidal ideation and behaviors in relation to having sufferen from the COVID-19 infection.

**Suicidal behavior**		**Total**		**Ever infected**			**Actually infected**		
	***n***	**Frec**.	**%**	**Yes**	**No**	**OR (95% CI)**	**Yes**	**No**	**OR (95% CI)**
1. Wish to be death	656	262	39.9	48.4	38	1.53 (1.03–2.27)^*^	42.9	39.9	1.12 (0.25–5.08)
2. Unspecific suicidal ideation	653	199	30.5	36.3	29.1	1.38 (0.91–2.09)	14.3	30.7	0.37 (0.045–3.15)
3. Suicidal ideation with any method	656	195	29.7	36.3	28.2	1.45 (0.96–2.19)	14.3	29.9	0.39 (0.04–3.26)
4. Suicidal ideation without a specific plan	656	159	24.2	27.4	23.5	1.23 (0.79–1.91)	28.6	24.2	1.25 (0.241–6.52)
5. Suicidal ideation with a specific plan	657	54	8.2	7.3	8.4	0.85 (0.40–1.80)	28.6	8	4.60 (0.87–24.29)
6. Previous suicidal attempts	656	120	18.2	19.5	18	1.10 (0.67–1.81)	28.6	18.2	1.80 (0.34–9.39)
7. Suicidal attempts in the last 3 months	120	48	40	33.3	41.7	0.70 (0.27–1.79)	0	40.7	1.02 (0.98–1.07)
8. Previous suicidal ideation or attempts	653	276	42.3	48.8	40.8	1.38 (0.93–2.05)	42.9	42.3	1.02 (0.25–4.61)
9. Non-specific preparatory behavior	626	86	13.7	14.2	13.6	1.04 (0.59–1.85)	0	13.9	NA
10. Preparatory behavior with a specific plan	629	64	10.2	12.4	9.6	1.32 (0.71–2.45)	14.3	10.1	1.47 (0.17–12.48)
11. Actual suicide attempt	631	84	13.3	14.2	13.1	1.09 (0.61–1.94)	0	13.5	NA

Odds ratio, calculated for 95% certainty. When the confidence interval did not include the value 1.

* p ≤ 0.05.

### Association between suicidal ideation and the consequences of COVID-19 for an acquaintance

When comparing the rates of suicidal indicators between the group of participants with a known person infected vs. “not” and a known dead person vs. “not,” the odds ratio (OR) was calculated to quantify the association between the possible risk factor and the percentage of each suicidal indicator, and it was considered statistically significant when the 95% CI did not include the value of 1; this identified the groups at risk. Table 5 shows the association between suicidal ideation and the consequences of COVID-19 for an acquaintance. In that sense, when participants had an “infected acquaintance,” it was identified as a risk factor, leading to significant values in the following suicide indicators: “wish to be dead” (OR = 1.71, 95% CI = 1.10–2.66); “unspecific suicidal ideation” (OR = 1.75, 95% CI = 1.07–2.85); “suicidal ideation with any method” (OR = 2.01, 95% CI = 1.20–3.34); “suicidal ideation without a specific plan” (OR = 1.80, 95% CI = 1.05–3.09); and “previous suicidal ideation or attempts” (OR = 1.57, 95% CI = 1.02–2.42). Further, it highlighted that knowing someone who died from COVID-19 was not associated with any suicidal indicators.

**Table 5:** Association between suicidal ideation and behavior and the consequences of COVID-19 for an acquaintance.

**Suicidal behavior**		**Total**		**Known person infected**			**Known person death**		
	***n***	**Frec**.	**%**	**Yes**	**No**	**OR (95% CI)**	**Yes**	**No**	**OR (95% CI)**
1. Wish to be dead	656	262	39.9	42	29.7	1.71 (1.10–2.66)^*^	40	39.9	1.00 (0.73–1.37)
2. Unspecific suicidal ideation	653	199	30.5	32.3	21.4	1.75 (1.07–2.85)^*^	29.7	31.4	0.92 (0.66–1.28)
3. Suicidal ideation with any method	656	195	29.7	31.9	18.9	2.01 (1.20–3.34)^**^	28.1	31.6	0.84 (0.60–1.18)
4. Suicidal ideation without a specific plan	656	159	24.2	25.9	16.2	1.80 (1.05–3.09)^*^	23.9	24.7	0.95 (0.67–1.37)
5. Suicidal ideation with a specific plan	657	54	8.2	8.6	6.3	1.41 (0.62–3.21)	7.8	8.8	0.87 (0.50–1.53)
6. Previous suicidal attempts	656	120	18.2	19.1	14.3	1.41 (0.80–2.51)	19.2	17.2	1.14 (0.76–1.71)
7. Suicidal attempts in the last 3 months	120	48	40	42.3	25	2.20 (0.66–7.27)	44.9	33.3	1.63 (0.77–3.45)
8. Previous suicidal ideation or attempts	653	276	42.3	44.1	33.3	1.57 (1.02–2.42)^*^	41.1	43.7	0.89 (0.65–1.22)
9. Non-specific preparatory behavior	626	86	13.7	14.4	10.4	1.45 (0.74–2.84)	12.5	15.3	0.78 (0.50–1.24)
10. Preparatory behavior with a specific plan	629	64	10.2	10.9	6.5	1.77 (0.78–4.00)	10.1	10.3	0.97 (0.58–1.64)
11. Actual suicide attempt	631	84	13.3	14.1	9.3	1.59 (0.79–3.19)	14.3	12.1	1.21 (0.75–1.93)

Odds ratio, calculated for 95% certainty. When the confidence interval did not include the value 1.

* p ≤ 0.05;

** p ≤ 0.01.

### Suicidal ideation in relation to confinement days

Table 6 shows the rates of suicidal indicators in relation to confinement. First, we compared the rates of suicidal indicators between the group of participants who “had been in confinement” vs. those who “had not.” Subsequently, to contrast the risk of suicidal ideas and behavior associated with the time of isolation, participants were re-categorized into two groups, first category where for participants who did not stayed in quarantine or who keep it for less than 40 days. Second category for those in self isolation for more than 40 days. The odds ratio (OR) was calculated to quantify the association between the possible risk factor and the percentage of each suicidal indicator. It was considered statistically significant when the 95% CI did not include the value of 1 to identify the groups at risk. The results showed that the group “in confinement” was a risk factor associated with the “desire to be dead” (OR = 1.81, 95% CI = 1.18–2.78) and “previous suicidal ideation or attempts” (OR = 1.97, 95% CI = 1.28–3.03). On the other hand, those who lasted “in confinement for more than 40 days” represented a higher risk of “wishing to be dead” (OR = 0.59, 95% CI = 0.43–0.818), “non-specific suicidal ideas” (OR = 0.66, 95% CI = 0.47–0.93), “suicidal ideation without a specific plan” (OR = 0.63, 95% CI = 0.44–0.91), “previous suicidal ideation or attempts” (OR = 0.55, 95% CI = 0.40–0.75), and “non-specific preparatory behavior” (OR = 0.57, 95% CI = 0.36–0.91).

**Table 6:** Association between suicidal ideation and behavior in relation to confinement time.

**Suicidal behavior**		**Total**		**In confinement**			**Confinement** > **40 days**		
	***n***	**Frec**.	**%**	**Yes**	**No**	**OR (95% CI)**	**Yes**	**No**	**OR (95% CI)**
1. Wish to be dead	656	262	39.9	42.4	28.9	1.81 (1.18–2.78)^**^	46.2	33.8	0.59 (0.43–0.818)^***^
2. Unspecific suicidal ideation	653	199	30.5	31.4	26.3	1.28 (0.82–2.01)	34.8	26.2	0.66 (0.47–0.93)^**^
3. Suicidal ideation with any method	656	195	29.7	30.5	26.4	1.21 (0.78–1.90)	30.9	28.6	0.89 (0.64–1.25)
4. Suicidal ideation without a specific plan	656	159	24.2	25.8	17.4	1.65 (0.99–2.75)	28.4	20.2	0.63 (0.44–0.91)^**^
5. Suicidal ideation with a specific plan	657	54	8.2	8.2	8.3	0.99 (0.48–2.03)	9.2	7.2	0.76 (0.43–1.34)
6. Previous suicidal attempts	656	120	18.2	17.8	20.7	0.82 (0.50–1.35)	20.6	16	0.73 (0.49–1.09)
7. Suicidal attempts in the last 3 months	120	48	40	44.2	24	2.50 (0.92–6.84)	46.3	32.1	0.54 (0.25–1.16)
8. Previous suicidal ideation or attempts	653	276	42.3	45.1	29.4	1.97 (1.28–3.03)^**^	49.5	35.2	0.55 (0.40–0.75)^***^
9. Non-specific preparatory behavior	626	86	13.7	14.2	11.7	1.25 (0.68–2.31)	17.1	10.6	0.57 (0.36–0.91)^**^
10. Preparatory behavior with a specific plan	629	64	10.2	10	11	0.89 (0.47–1.70)	11.4	9	0.76 (0.45–0.1.28)
11. Actual suicide attempt	631	84	13.3	13.7	11.8	1.18 (0.64–2.19)	15.9	10.9	0.64 (0.40–1.03)

Odds ratio, calculated for 95% certainty. When the confidence interval did not include the value 1.

** p ≤ 0.01;

*** p ≤ 0.001.

## Discussion

Before the pandemic, suicide was already considered a global public health problem, and researchers developed theories to explain the factors that contributed to or mitigated this risk. For this reason, the present work examined ***the prevalence of suicidal ideation and behavior related to COVID-19 in the population of the northeastern border region of Mexico***.

Our first specific objective was **to describe the prevalence of suicidal ideation and behavior during the COVID-19 pandemic in the population of the northeastern border region of Mexico**. Our results indicated that the most frequent indicators of suicidal ideation were “wish to be dead,” reported by 39.9% of participants, “unspecific suicidal ideation,” reported by 30.5%, “suicidal ideation with any method,” reported by 29.7%, and “suicidal ideation without a specific plan,” reported by almost a quarter of the participants (24.2%); less frequent were the indicators of suicidal behaviors such as “non-specific preparatory behavior,” reported by 13.7% of participants; “actual suicide attempts,” reported by 13.3%, and “non-specific preparatory behavior,” reported by 10.2%. Nevertheless, given that those are considered to be at a higher risk of committing suicide, we considered alert signs, especially considering that nearly a fifth of participants (18.2%) reported “previous suicidal attempts,” of whom 40% had “suicidal attempts in the last 3 months” and 42.3% had a history of “previous suicidal ideation or attempts.” The literature referred to the fact that, in terms of sex, women presented a 20–30% higher risk of manifesting suicidal ideation and behavior (Monteith et al., 2021; Nomura et al., 2021). The results of our study (Table 2) provide evidence for this situation, given that in most of the questions, women presented a higher risk of suicidal ideation and behavior during the COVID-19 contingency period. In a similar vein, some authors pointed out that, among their participants, suicidal thoughts were found to be in slightly more than half of the female participants (Fitzpatrick et al., 2020).

This statement is likely due to the expression of greater emotional sensitivity and because they manifest greater vulnerability to stressors that trigger negative psychological consequences, such as the death of family or friends (Hermosillo-de-la-Torre et al., 2021; Rahman et al., 2021).

The second specific objective was **to analyze the suicidal ideation and behavior associated with risk factors for the contagion of COVID-19**. In the present study, the questions addressed personal infection or that of any known person with COVID-19; in this sense, the results did not show significant sex differences. It is essential to point out that there were significant data regarding the period of confinement, suggesting that 86.2% of the women and 70.6% of the men maintained isolation (Table 3). It was observed that 61.3% of the women maintained confinement for periods longer than 40 days, compared to only 51.8% of the men. Although it is impossible to generalize, these results could be related to gender roles and the distribution of family activities. This situation becomes complex, as it requires studying the individual's identity, social role, and social relationships, which could influence the exposure and contagion of infectious diseases such as COVID-19 (Ya'qoub et al., 2021). The role of economic breadwinner falls on men rather than women, so men may need to continue to go out to work while women stay at home to care for children and the household. However, the probability of personal and family contagion is the same for both sexes since the person who needs to go out could infect the rest of the family.

The COVID-19 pandemic is exacerbating sex discrimination in the current society, which represents a complicated situation for the administration of public policies, given that, globally, only two-thirds of the data on COVID-19 infections have not been classified by sex (Nordhues et al., 2021). It is important to look at the data on infection analysis and to consider the vulnerability of individuals in terms of their level of health and their classification according to sex (Ya'qoub et al., 2021). While it is true that women have a higher perception of the threat of COVID-19, they are also the ones who are exposed to a higher level of infection, only when considering that healthcare workers are composed of at least 70% of the female population (Hermosillo-de-la-Torre et al., 2021; Nordhues et al., 2021). However, global figures indicate that male patients present a higher number of complications and deaths from COVID-19 (Ortolan et al., 2020).

Studies exploring gender differences in the association between stressful events and psychological diseases suggest that it is likely that there are gender differences in the association between stressful life events and psychological diseases such as psychosis, depression, and anxiety (Kendler et al., 2001; Herbison et al., 2017; Mansueto and Faravelli, 2022).

With regard to the association between suicidal ideation and behaviors in relation to personal COVID-19 contagion, the findings of our work showed that 48.4% of the participants that have been infected commonly presented “the wish to be dead.” This shows that people who had COVID-19 also had high levels of stress, anxiety, and depression that went unnoticed.

It is important to consider that the pandemic negatively affects individuals' mental health and emotional wellbeing, which, in the case of poor psychosocial and mental functioning, increases the risk of contracting COVID-19 disease (Elbogen et al., 2021). Based on this premise, the rates of infection, hospitalization, and death due to COVID-19 may have a higher prevalence of suicidal ideation and behavior in areas where the disease is more prevalent (Bonsaksen et al., 2021).

In other words, concerns arising from possible complications in the case of contracting the virus are associated with increased suicidal thoughts and behaviors (Bonsaksen et al., 2021). Those who confirmed COVID-19 infection were more likely to have negative mental health aspects, such as suicidal ideation and behavior of those who only suspected infection (Shi et al., 2021).

When assessing the association between suicidal ideation and the consequences of COVID-19 for an acquaintance, our results demonstrated the presence of an acquaintance infected with COVID-19 disease as a risk factor for the presence of suicidal ideation and behavior. In the following order, “having suicidal thoughts or attempts in other periods of his or her life; the wish to be dead; unspecific suicidal ideas; suicidal ideas with a method; and suicidal ideation without a specific plan” were the most significant items. It is likely that such thoughts and ideas result from excessive information shared by friends and family, mainly due to dissemination through social networks.

The presence of suicidal ideation and behaviors increases when a group of people or a certain population faces a disease outbreak that may affect them (Rahman et al., 2021). The impact of COVID-19-related experiences may be associated with suicidal ideation and contribute to suicidal behaviors, mainly in the wake of recent events (Ammerman et al., 2021). Moreover, recent studies suggest that psychological diseases related to the COVID-19 pandemic stressors may be exacerbated by COVID-19 dysfunctional coping strategies (e.g., the COVID-19 anxiety syndrome, worry, and rumination) in response to the fear or threat of the COVID-19 pandemic itself (Nikčević and Spada, 2020; Nikčević et al., 2021; Mansueto et al., 2022).

Our third objective was **to analyze the suicidal ideation and behavior associated with confinement during the COVID-19 pandemic**; our results indicated that living in confinement for more than 40 days represents a risk factor for the presence of suicidal ideation and behavior. The desire to be dead, unspecific suicidal ideas, and suicidal ideation without a specific plan, as well as suicidal thoughts and attempts at different periods in their lives, were the most significant issues. This implies that those who remained in confinement for long periods saw an increase in their stress and anxiety, affecting their mental health and even provoking negative thoughts of attempting suicide. Our results are consistent with the results revealed by Nikčević and Spada (2020), who pointed out that perceived social support and greater perceived loneliness were significantly related to greater thoughts of suicide/self-harm, which is consistent with the interpersonal theory of suicide (Joiner, 2007). In this case, to reduce the risk associated with confinement, despite its name, social distancing requires physical space between people, not social distance. Efforts can be made to stay connected and maintain meaningful relationships by telephone or video, especially among individuals with substantial risk factors for suicide (Reger et al., 2020).

Our results pointed out the risk related to the population with preexisting suicidal behaviors. It is probable that stress related to confinement could be a factor that decreases the coping mechanisms of participants and that adverse factors are summarized in the history of life adversity events related to a higher risk of suicidal behavior (Herbison et al., 2017; Kahil et al., 2021).

Our findings corroborate those of earlier studies, which found that family unity was strengthened by people staying at home and adhering to the restriction measures (Ammerman et al., 2021; Anzai et al., 2021). On the other hand, staying at home has led to a mental burden that increases levels of depression and anxiety in young populations, making it a risk factor for suicidal ideation and behavior (Sayeed et al., 2020; Nomura et al., 2021; Rahman et al., 2021).

In conclusion, our study results support what has been stated in previous studies about the chronicity of stressful events and different mental health outcomes related to exposure related to temporality, chronicity, and gender. For example, as stated by Herbison et al. (2017), in women, medium-to-high chronic stress exposure or exposure during puberty/adolescence predicted depression and anxiety symptoms, while low or reduced stress exposure over the life course did not. Further, for women, postnatal stress trajectory was more important than prenatal stress in predicting depression/anxiety symptoms. Meanwhile, high stress early in pregnancy was associated with depression and anxiety symptoms in men, regardless of postnatal stress trajectory.

The higher rates of suicidal ideation and behavior related to the risk factors of being a woman, having a previous suicide attempt, knowing a person infected with COVID-19, and having the time of lockdown reported in our study are risk factors that should be considered in clinical practice. Our results support studies that have reported the link between multiple stressors such as social isolation, stress, financial strain, and suicide (Elbogen et al., 2021). The consideration of sex and age of participants could play a role in what are considered risk factors for suicide, as stated by Elbogen et al. (2021) when examining thoughts of suicide/self-harm. They found significant interactions between financial strain and being male, above the median age of 35 years, non-White, and Hispanic. Those factors could be related to financial strain and the roles of family economic providers.

Since the beginning of the pandemic, experts and early-career psychiatrists have called for global action to prevent suicide during this outbreak. Clinicians could benefit greatly from an accurate diagnosis that takes into account the risk factors for suicidal behavior as well as the underlying mental disorders involved (Nikčević and Spada, 2020; Mansueto et al., 2022). An adequate diagnosis and considering the risk factors for suicidal behavior and the underlying mental resources involved could be of great help to clinicians to ensure the quality of attention.

## Conclusion

The present study aimed to examine the prevalence of suicidal ideation and behavior related to COVID-19 in the population of the northeastern border region of Mexico. To this end, our main findings showed that 39.9% of participants wished they were dead. A total of 18.2% of participants had suicidal behaviors before the COVID-19 pandemic, of which 40% had suicidal behaviors during the 3 months before the study during the pandemic. Regarding differentiation by sex, we found significant differences in almost all suicidal ideation and behavior indicators before and during the pandemic, with women representing a higher risk. COVID-19 infected 18.8% of the participants, 82.9% knew someone infected, and 54.8% knew someone who died from COVID-19, without significant differences between sexes. Further, 81.6% of participants reported having lived in confinement, finding significant differences between the sexes, with this rate being higher in women (86.2%) than in men (70.6%). Being infected with COVID-19 is associated with a higher risk of wishing to be dead but not with the rest of the suicidal ideas and behaviors. Knowing a person infected with COVID-19 was associated with several suicidal thoughts and with previous suicidal ideas and attempts. While knowing a person who died from COVID-19 was not a risk factor for any of them, staying in confinement was associated with a higher risk of the wish to be dead, occurring in 42.4% of the participants who had maintained confinement vs. 28.9% of those who had not, and it was also associated with suicidal ideation and behaviors in other periods of life. Confinement time >40 days was a risk factor for several indicators of suicidal ideas and behaviors compared to not having been confined or kept in isolation for <40 days.

It will be complicated to establish definitive conclusions, given the temporality of the pandemic; however, future lines of research related to economic situations, employment, level of health, alcohol consumption, and drug use, among others, could be established.

Intervention programs and strategies are required to minimize the suicide risk statistics, especially in light of the pandemic's influence and the fact that the consequences on mental health may endure well beyond the duration of the pandemic. In clinical practice, given that the pandemic poses unprecedented risks, the clinical diagnosis tools, and intervention programs should be tailored to address specific situations. When facing cases of suicide-risk patients, recently created instruments and intervention proposals that consider disorders such as COVID-19 fear and anxiety syndrome are recommended. In the post-COVID-19 era, dissemination and training programs for clinicians will be essential.

It will be essential to develop protective, self-care, and social support skills, especially in those individuals who present risk factors for suicidal behavior identified in this study: women, those who knew a COVID-19-infected patient, those who were confined for longer than 40 days, and those who had a history of suicidal ideas and behaviors. It will also be important to take advantage of recent technological advances (e.g., videoconferencing), which could facilitate progress and make confinement and social distancing more bearable.

The authors consider that this study's main limitation consists of the evolution of the COVID-19 disease, which causes changes in daily life. Moreover, the data collection in this study was carried out in the early stages of the pandemic. In addition, given the lack of control and the nature of the procedure used—a convenience sample assessed by a self-reported online survey—there are no other control measures such as laboratory or result tests for COVID-19 contagion. Furthermore, since no instrument assessed suicidal risk during the COVID-19 health emergency in the Mexican population, the instrument used was adapted for the study. The authors acknowledge that their study's findings are restricted to those living in Mexico's northwestern border state, where they had internet, computer, or smartphone access necessary to complete the survey, and where the survey was initially disseminated through a variety of channels related to higher education. To fill these gaps and generalize results, future studies should consider increasing the size of the sample, such as including populations from different places in Mexico and other sociodemographic characteristics that have not been included in this study, such as illiterate people.

## Data availability statement

The raw data supporting the conclusions of this article will be made available by the authors, without undue reservation.

## Ethics statement

The studies involving human participants were reviewed and approved by Postgraduate and Research Committee at Universidad Autonoma de Tamaulipas. The patients/participants provided their written informed consent to participate in this study.

## Author contributions

KV conceived and designed the project, authored and integrated the reviewed drafts of the paper, approved the final draft, and substantially contributed to the interpretation of the data. FP authored and integrated the reviewed drafts of the paper, approved the final draft, and substantially contributed to the interpretation of the data. BZ, CV, IH, and CL authored and reviewed drafts of the paper, approved the final draft, and substantially contributed to the interpretation of the data. All authors contributed to the article and approved the submitted version.

## References

[B1] Al-Halabi S. Saiza P. A. Buron P. Garrido M. Benabarre A. Jimenez E.. (2016). Validation of Spanish version of the Columbia-Suicide Severity Rating Scale (C-SSRS). Rev. Psiquiatr. Salud Ment. 9, 134–142. 10.1016/j.rpsm.2016.02.002 27158026

[B2] Ammerman B. A. Burke T. A. Jacobucci R. McClure K. (2021). Preliminary investigation of the association between COVID-19 and suicidal thoughts and behaviors in the U.S. J. Psychiatr. Res. 134, 32–38. 10.1016/j.jpsychires.2020.12.037 33360222

[B3] Anzai T. Fukui K. Ito T. Ito Y. Takahashi K. (2021). Excess mortality from suicide during the early COVID-19 pandemic period in Japan: a time-series modeling before the pandemic. J. Epidemiol. 31, 152–156. 10.2188/jea.JE20200443 33310986 PMC7813773

[B4] Aragón-Nogales R. Vargas-Almanza I. Miranda-Novales M. G. (2019). COVID-19 por SARS-CoV-2: La nueva emergencia de salud. Rev. Mex. Pediatr. 86, 213–218. 10.35366/91871

[B5] Bonsaksen T. Skogstad L. Heir T. Ekeberg Ø. Schou-Bredal I. Grimholt T. K. (2021). Suicide thoughts and attempts in the Norwegian general population during the early stage of the COVID-19 outbreak. Int. J. Environ. Res. Public Health 18, 4102. 10.3390/ijerph18084102 33924558 PMC8069206

[B6] Brailovskaia J. Cosci F. Mansueto G. Margraf J. (2021). The relationship between social media use, stress symptoms and burden caused by coronavirus (COVID-19) in Germany and Italy: a cross-sectional and longitudinal investigation. J. Affect. Disord. Rep. 3, 100067. 10.1016/j.jadr.2020.100067 35434690 PMC8995101

[B7] Brailovskaia J. Margraf J. (2020). Predicting adaptive and maladaptive responses to the Coronavirus (COVID-19) outbreak: A prospective longitudinal study. Int. J. Clin. Health Psychol. 20, 183–191. 10.1016/j.ijchp.2020.06.002 32837518 PMC7321043

[B8] Cheung T. Lam S. C. Lee P. H. Xiang Y. T. Yip P. S. F. The International Research Collaboration on COVID-19 (2021). Global imperative of suicidal ideation in 10 countries amid the COVID-19 pandemic. Front. Psychiatry 11, 588781. 10.3389/fpsyt.2020.588781 33519545 PMC7838349

[B9] Devitt P. (2020). Can we expect an increased suicide rate due to Covid-19? Ir. J. Psychol. Med. 37, 264–268. 10.1017/ipm.2020.46 32434598 PMC7287306

[B10] Elbogen E. B. Lanier M. Blakey S. M. Wagner H. R. Tsai J. (2021). Suicidal ideation and thoughts of self-harm during the COVID-19 pandemic: the role of COVID-19 related stress, social isolation, and financial strain. Depress. Anxiety 2021, da.23162. 10.1002/da.23162 33949747 PMC8239640

[B11] Fitzpatrick K. M. Harris C. Drawve G. (2020). How bad is it? Suicidality in the middle of the COVID-19 pandemic. Suicide Life-Threaten. Behav. 50, 1241–1249. 10.1111/sltb.12655 32589799 PMC7361329

[B12] Galea S. Merchant R. M. Lurie N. (2020). The mental health consequences of COVID-19 and physical distancing: The need for prevention and early intervention. JAMA Intern. Med. 180, 817–818. 10.1001/jamainternmed.2020.1562 32275292

[B13] Han J. McGillivray L. Wong Q. J. Werner-Seidler A. Wong I. Calear A.. (2020). A mobile health intervention (Lifebuoy app) to help young people manage suicidal thoughts: protocol for a mixed-methods randomized controlled trial. JMIR Res. Protoc. 9, e23655. 10.2196/23655 33107832 PMC7655466

[B14] Herbison C. E. Allen K. Robinson M. Newnham J. Pennell C. (2017). The impact of life stress on adult depression and anxiety is dependent on gender and timing of exposure. Develop. Psychopathol. 29, 1443–1454. 10.1017/S0954579417000372 28397629

[B15] Hermosillo-de-la-Torre A. E. Arteaga-de-Luna S. M. Acevedo-Rojas D. L. Juárez-Loya A. Jiménez-Tapia J. A. Pedroza-Cabrera F. J.. (2021). Psychosocial correlates of suicidal behavior among adolescents under confinement due to the COVID-19 Pandemic in Aguascalientes, Mexico: a cross-sectional population survey. Int. J. Environ. Res. Public Health 18, 4977. 10.3390/ijerph18094977 34067094 PMC8124170

[B16] Huang Y. Su X. Si M. Xiao W. Wang H. Wang W.. (2021). The impacts of coping style and perceived social support on the mental health of undergraduate students during the early phases of the COVID-19 pandemic in China: A multicenter survey. BMC Psychiatry. 21, 530. 10.1186/s12888-021-03546-y 34706690 PMC8549419

[B17] INEGI (2020). Estadísticas a propósito del ‘Día mundial para la prevención del suicidio'. Datos nacionales. Comunicado de Prensa No. 422/20; p. 7. Instituto Nacional de Estadística y Geografía. Available online at: https://www.inegi.org.mx/contenidos/saladeprensa/aproposito/2020/suicidios2020_Nal.pdf (accessed December 5, 2022).

[B18] Joiner T. (2007). Why People Die by Suicide. Harvard University Press.

[B19] Kahil K. Cheaito M. A. El Hayek R. Nofal M. El Halabi S. Kudva K. G.. (2021). Suicide during COVID-19 and other major international respiratory outbreaks: a systematic review. Asian J. Psychiatr. 56, 102509. 10.1016/j.ajp.2020.102509 33418284 PMC7764387

[B20] Kar S. K. Yasir Arafat S. M. Kabir R. Sharma P. Saxena S. K. (2020). Coping with mental health challenges during COVID-19, in Coronavirus Disease 2019 (COVID-19): Epidemiology, Pathogenesis, Diagnosis, and Therapeutics, ed Saxena S. (Singapore: Springer), 199–213. 10.1007/978-981-15-4814-7_16

[B21] Kendler K. S. Gardner C. O. Neale M. C. Prescott C. A. (2001). Genetic risk factors for major depression in men and women: Similar or different heritabilities and same or partly distinct genes? Psychol. Med. 31, 605–616. 10.1017/s0033291701003907 11352363

[B22] Kiuchi K. Kishi K. Araki K. (2020). A foundational assessment of the effects of the spread of COVID-19 virus infection and related activity restrictions on mental and physical health, psychological distress, and suicidal ideation in Japan. Asia Pac. J. Public Health 32, 4. 10.1177/10105395209654 33070620

[B23] Lee S. W. Lee J. Moon S. Y. Jin H. Y. Yang J. M. Ogino S.. (2022). Physical activity and the risk of SARS-CoV-2 infection, severe COVID-19 illness and COVID-19 related mortality in South Korea: A nationwide cohort study. Br. J. Sports Med. 56, 901–912. 10.1136/bjsports-2021-104203 34301715

[B24] Longobardi C. Morese R. Fabris M. A. (2020). COVID-19 emergency: social distancing and social exclusion as risks for suicide ideation and attempts in adolescents. Front. Psychol. 11, 551113. 10.3389/fpsyg.2020.551113 33329182 PMC7710515

[B25] Mansueto G. Faravelli C. (2022). Stressful life events and psychosis: gender differences. Stress Health 38, 19–30. 10.1002/smi.3067 33973342

[B26] Mansueto G. Lopes F. L. Grassi L. Cosci F. (2021). Impact of COVID-19 outbreak on Italian healthcare workers versus general population: results from an online survey. Clin. Psychol. Psychother. 28, 1334–1345. 10.1002/cpp.2644 34255890 PMC8426916

[B27] Mansueto G. Palmieri S. Marino C. Caselli G. Sassaroli S. Ruggiero G. M.. (2022). The Italian COVID-19 Anxiety Syndrome Scale: investigation of the COVID-19 anxiety syndrome and its association with psychological symptoms in an Italian population. Clin. Psychol. Psychother. 29, 1791–1990. 10.1002/cpp.2767 35771682 PMC9350361

[B28] Monteith L. L. Holliday R. Hoffmire C. A. (2021). Understanding women's risk for suicide during the COVID-19 pandemic: a call to action. Psychiatry Res. 295, 113621. 10.1016/j.psychres.2020.113621 33302132

[B29] Nikčević A. V. Marino C. Kolubinski D. C. Leach D. Spada M. M. (2021). Modelling the contribution of the Big Five personality traits, health anxiety, and COVID-19 psychological distress to generalised anxiety and depressive symptoms during the COVID-19 pandemic. J. Affect. Disord. 279, 578–584. 10.1016/j.jad.2020.10.053 33152562 PMC7598311

[B30] Nikčević A. V. Spada M. M. (2020). The COVID-19 anxiety syndrome scale: development and psychometric properties. Psychiatry Res. 292, 113322. 10.1016/j.psychres.2020.113322 32736267 PMC7375349

[B31] Nomura K. Minamizono S. Maeda E. Kim R. Iwata T. Hirayama J.. (2021). Cross-sectional survey of depressive symptoms and suicide-related ideation at a Japanese national university during the COVID-19 stay-home order. Environ. Health Prev. Med. 26, 30. 10.1186/s12199-021-00953-1 33673802 PMC7934991

[B32] Nordhues H. C. Bhagra A. Stroud N. N. Vencill J. A. Kuhle C. L. (2021). COVID-19 gender disparities and mitigation recommendations: a narrative review. Mayo Clin. Proceed. 96, 1907–1920. 10.1016/j.mayocp.2021.04.009 34218863 PMC8057762

[B33] Odone A. Lugo A. Amerio A. Borroni E. Bosetti C. Carreras G.. (2020). COVID-19 lockdown impact on lifestyle habits of Italian adults. Acta Bio-Med. Atenei Parmensis 91, 87–89. 10.23750/abm.v91i9-S.10122 32701921 PMC8023096

[B34] Ortolan A. Lorenzin M. Felicetti M. Doria A. Ramonda R. (2020). Does gender influence clinical expression and disease outcomes in COVID-19? A systematic review and meta-analysis. Int. J. Infect. Disord. 99, 496–504. 10.1016/j.ijid.2020.07.076 32800858 PMC7422797

[B35] Pathirathna M. L. Nandasena H. M. R. K. G. Atapattu A. M. M. P. Weerasekara I. (2022). Impact of the COVID-19 pandemic on suicidal attempts and death rates: A systematic review. BMC Psychiatry. 22, 506. 10.1186/s12888-022-04158-w 35902951 PMC9331016

[B36] Penninx B. W. J. H. Eikelenboom M. Giltay E. J. van Hemert A. M. Riese H. Schoevers R. A.. (2021). Cohort profile of the longitudinal Netherlands Study of Depression and Anxiety (NESDA) on etiology, course and consequences of depressive and anxiety disorders. J. Affect. Disord. 287, 69–77. 10.1016/j.jad.2021.03.026 33773360

[B37] Posner K. Brent D. Lucas C. Gould M. Stanley B. Brown G.. (2010). Escala Columbia para evaluar la seriedad de la ideación suicida (C-SSRS). New York State Psychiatric Institute. Available online at: https://cssrs.columbia.edu/wp-content/uploads/C-SSRS-CognitivelyImpared-SinceLastContact-US-spanish.pdf (accessed December 5, 2022).

[B38] Posner K. Brown G. K. Stanley B. Brent D. A. Yershova K. V. Oquendo M. A.. (2011). The Columbia–Suicide severity rating scale: initial validity and internal consistency findings from three multisite studies with adolescents and adults. Am. J. Psychiatry 168, 1266–1277. 10.1176/appi.ajp.2011.10111704 22193671 PMC3893686

[B39] Rahman H. A. Amornsriwatanakul A. Abdul-Mumin K. H. Agustiningsih D. Chaiyasong S. Chia M.. (2022). Prevalence of health-risk behaviors and mental well-being of ASEAN University students in COVID-19 pandemic. Int. J. Environ. Res. Public Health 19, 8528. 10.3390/ijerph19148528 35886375 PMC9320216

[B40] Rahman M.d. E. Zubayer A. A. Al Mazid Bhuiyan Md. R. Jobe M. C. Ahsan Khan Md. K. (2021). Suicidal behaviors and suicide risk among Bangladeshi people during the COVID-19 pandemic: an online cross-sectional survey. Heliyon 7, e05937. 10.1016/j.heliyon.2021.e05937 33615003 PMC7879153

[B41] Reger M. A. Stanley I. H. Joiner T. E. (2020). Suicide mortality and coronavirus disease 2019—a perfect storm? JAMA Psychiatry 77, 1093. 10.1001/jamapsychiatry.2020.1060 32275300

[B42] Salari N. Hosseinian-Far A. Jalali R. Vaisi-Raygani A. Rasoulpoor S. Mohammadi M.. (2020). Prevalence of stress, anxiety, depression among the general population during the COVID-19 pandemic: A systematic review and meta-analysis. Glob. Health. 16, 57. 10.1186/s12992-020-00589-w 32631403 PMC7338126

[B43] Saricali M. Satici S. A. Satici B. Gocet-Tekin E. Griffiths M. D. (2020). Fear of COVID-19, mindfulness, humor, and hopelessness: a multiple mediation analysis. Int. J. Ment. Health Addict. 20, 2151–2164. 10.1007/s11469-020-00419-5 33230394 PMC7676415

[B44] Sayeed A. Kundu S. Banna M.d,. H. A Hasan M. T. Begum M. R.. (2020). Mental health outcomes during the COVID-19 and perceptions towards the pandemic: findings from a cross sectional study among Bangladeshi students. Child. Youth Serv. Rev. 119, 105658. 10.1016/j.childyouth.2020.105658 33518861 PMC7833817

[B45] Serafini G. Pompili M. Innamorati M. Gentile G. Borro M. Lamis D. A.. (2012). Gene variants with suicidal risk in a sample of subjects with chronic migraine and affective temperamental dysregulation. Eur. Rev. Med. Pharmacol. Sci. 16, 1389–1398. 23104655

[B46] Shi L. Que J.-Y. Lu Z.-A. Gong Y.-M. Liu L. Wang Y.-H.. (2021). Prevalence and correlates of suicidal ideation among the general population in China during the COVID-19 pandemic. Eur. Psychiatry 64, e18. 10.1192/j.eurpsy.2021.5 33686933 PMC7943957

[B47] Smalley C. M. Malone D. A. Meldon S. W. Borden B. L. Simon E. L. Muir M. R.. (2021). The impact of COVID-19 on suicidal ideation and alcohol presentations to emergency departments in a large healthcare system. Am. J. Emerg. Med. 41, 237–238. 10.1016/j.ajem.2020.05.093 32505472 PMC7263212

[B48] World Health Organization (2020). WHO Coronavirus (COVID-19) Dashboard. Available online at: https://covid19.who.int (accessed December 5, 2022).

[B49] Ya'qoub L. Elgendy I. Y. Pepine C. J. (2021). Sex and gender differences in COVID-19: more to be learned! Am. Heart J. Plus: Cardiol. Res. Pract. 3, 100011. 10.1016/j.ahjo.2021.100011 34169297 PMC8045422

[B50] Zhang C. Ye M. Fu Y. Yang M. Luo F. Yuan J.. (2020). The psychological impact of the COVID-19 pandemic on teenagers in China. J. Adolesc. Health 67, 747–755. 10.1016/j.jadohealth.2020.08.026 33041204 PMC7543885

